# A risk prediction model for type 2 diabetes mellitus complicated with retinopathy based on machine learning and its application in health management

**DOI:** 10.3389/fmed.2023.1136653

**Published:** 2023-04-27

**Authors:** Hong Pan, Jijia Sun, Xin Luo, Heling Ai, Jing Zeng, Rong Shi, An Zhang

**Affiliations:** ^1^Department of Health Management, School of Public Health, Shanghai University of Traditional Chinese Medicine, Shanghai, China; ^2^Department of Mathematics and Physics, School of Pharmacy, Shanghai University of Traditional Chinese Medicine, Shanghai, China; ^3^Department of Public Utilities Management, School of Public Health, Shanghai University of Traditional Chinese Medicine, Shanghai, China

**Keywords:** diabetic retinopathy, least absolute shrinkage selection operator (LASSO) model, random forest recursive feature elimination (RF-RFE) algorithm, extreme gradient boosting (XGBoost) algorithm, backpropagation neural network (BPNN) model, nomogram

## Abstract

**Objective:**

This study aimed to establish a risk prediction model for diabetic retinopathy (DR) in the Chinese type 2 diabetes mellitus (T2DM) population using few inspection indicators and to propose suggestions for chronic disease management.

**Methods:**

This multi-centered retrospective cross-sectional study was conducted among 2,385 patients with T2DM. The predictors of the training set were, respectively, screened by extreme gradient boosting (XGBoost), a random forest recursive feature elimination (RF-RFE) algorithm, a backpropagation neural network (BPNN), and a least absolute shrinkage selection operator (LASSO) model. Model I, a prediction model, was established through multivariable logistic regression analysis based on the predictors repeated ≥3 times in the four screening methods. Logistic regression Model II built on the predictive factors in the previously released DR risk study was introduced into our current study to evaluate the model’s effectiveness. Nine evaluation indicators were used to compare the performance of the two prediction models, including the area under the receiver operating characteristic curve (AUROC), accuracy, precision, recall, F1 score, balanced accuracy, calibration curve, Hosmer-Lemeshow test, and Net Reclassification Index (NRI).

**Results:**

When including predictors, such as glycosylated hemoglobin A1c, disease course, postprandial blood glucose, age, systolic blood pressure, and albumin/urine creatinine ratio, multivariable logistic regression Model I demonstrated a better prediction ability than Model II. Model I revealed the highest AUROC (0.703), accuracy (0.796), precision (0.571), recall (0.035), F1 score (0.066), Hosmer-Lemeshow test (0.887), NRI (0.004), and balanced accuracy (0.514).

**Conclusion:**

We have built an accurate DR risk prediction model with fewer indicators for patients with T2DM. It can be used to predict the individualized risk of DR in China effectively. In addition, the model can provide powerful auxiliary technical support for the clinical and health management of patients with diabetes comorbidities.

## 1. Introduction

Diabetic retinopathy (DR) is one of the most common microvascular complications of diabetes. DR is a series of fundus diseases caused by retinal microvascular leakage and occlusion from chronic progressive diabetes (1–3). The prevalence of DR in type 2 diabetes mellitus (T2DM) patients is 22.27% (4). As the incidence of diabetes increases, the number of DR patients suffering from severe retinal damage with further complications, such as fluid exudation, bleeding, detachment, and eventually blindness, will increase to 160.50 million by 2045 worldwide (4, 5). In addition to destructive visual effects that may lead to inconveniences in mobility, a decline in quality of life, and depression, DR is also associated with a high risk of systemic vascular complications. This increases the mortality risk and places a heavy economic burden on the medical system (6–8). The high prevalence and severity of DR indicate a need for early DR screening. Due to the large number of patients requiring consultation and complex individual differences, it is unlikely all patients will receive an eye examination at an appropriate time. Thus, appropriate methods, such as building corresponding prediction models, will help to predict disease risks, and appropriate interventions will help to reduce the incidence rate of diseases (9–12).

With the advent of the era of artificial intelligence, machine learning methods, which include long short-term memory networks and random forest (RF), have been gradually introduced into a wide range of fields, such as planetscope nanosatellites image classification (13), automated weed detection system (14, 15), modeling of groundwater storage change (16–19), supervised image classification (20), and analysis of environmental factors (21), and have achieved good results. Therefore, machine learning methods have important applications in medical management, such as disease prediction.

Currently, machine learning methods are gradually being applied to disease prediction in DR (22–25). For example, 17 indicators, including age, fasting blood glucose (FBG), glycosylated hemoglobin A1c (HbA1c), and total cholesterol, have been used. Wanyue Li et al. built a DR risk prediction model based on the XGBoost algorithm, which has good comprehensive performance and high reliability regarding DR risk indicators (26). By selecting characteristic variables through minimum absolute contraction and least absolute shrinkage selection operator (LASSO) regression optimization and RF analysis, Hongyan Yang et al. constructed a corresponding logistic regression prediction model based on the data of 5900 T2DM patients. They analyzed the risks associated with DR (diabetes duration, diabetic neuropathy, diabetic nephropathy, diabetic foot, hyperlipidemia, hypoglycemic drugs, glycosylated albumin, and lactate dehydrogenase). The corresponding result can effectively identify and intervene in DR high-risk groups at an early stage (27). Li Yongsheng et al. used LASSO regression and multiple logistic regression analysis to select variables and establish a model containing indicators, such as diabetic peripheral neuropathy, age, neuropathies, high sensitivity lipoprotein (HDL), HbA1c, T2DM duration, and glycolytic serum protein. The model can predict the personalized risk of DR in patients in Xinjiang, China (28). At present, the gold standard for the diagnosis of diabetic peripheral neuropathy is nerve conduction studies; however, these are labor-intensive, time-consuming, expensive, and impractical (29). Diagnosing diabetic foot requires examination of both lower limb arteries and foot and ankle orthopedic examination by color Doppler ultrasound, which is time-consuming and costly (30). Therefore, it is difficult to obtain comprehensive prediction indicators.

We found that the current construction method of the DR risk prediction model is relatively single, the number of selected indicators is large, and some indicators are not easy to obtain. Therefore, there is a need for a DR risk prediction model that contains fewer indicators and is thus more accessible. Because different screening methods have different characteristics, some key risk factors may be neglected, or some features with poor prediction ability may be included in the screening process (25, 31–34). For example, RF uses an integrated algorithm, which is better than most single algorithms in its accuracy, but will overfit the classification problem with high noise (35, 36). LASSO regression analysis can reduce dimensions by compressing high-dimensional variables. However, when there is a strong correlation between variables, only one variable is randomly selected, and the more important variable cannot be distinguished (31, 37). Therefore, in this study, we aimed to use four machine learning methods, including LASSO regression analysis, RF, XGBoost, and backpropagation neural network (BPNN), that are more advanced in building disease prediction models to screen characteristic indicators (38). To compare the advantages and disadvantages of using multiple and single machine learning methods to screen features and build models, we aim to introduce DR risk prediction models built by other scholars for multi-dimensional comparison. Considering the different sensitivities of different populations in different regions to the disease model, the DR risk assessment model for T2DM patients constructed by other scholars was selected based on the same population in the same region (Shanghai) for comparison in 9 dimensions: the receiver operating characteristic curve (ROC), accuracy, precision, recall, machine learning evaluation score (F1 score), balanced accuracy, calibration curve, Hosmer-Lemeshow test, and Net Reclamation Index (NRI).

Thus, this study aimed to screen fewer characteristic indicators through various machine learning methods and establish a DR risk prediction model with higher prediction ability. To reduce the deviation in the model construction process, we used multi-dimensional evaluation indicators to comprehensively evaluate the model’s performance and improve the model’s universality. In addition, we aimed to provide corresponding decision charts to help medical workers quantify the individual risk of T2DM, provide a reference for medical workers to prevent and control diabetes, and further improve the community health management model. Intervention through community chronic disease management can effectively reduce and stabilize patients’ disease development, improve DR and self-management awareness, and improve treatment compliance.

## 2. Materials and methods

### 2.1. Study design

This cross-sectional survey was conducted in six community health service centers in Yangpu District and Pudong New District, including Huamu, Jinyang, Sanlin, Yinhang, Siping, and Daqiao communities in Shanghai, from October 2014 to April 2015. Most T2DM patients with more severe DR (mainly proliferative DR) were advised to undergo invasive clinical treatment (such as photocoagulation, vitrectomy, or intraocular drug injection) to prevent DR aggravation and improve visual function. However, since the above treatment would affect the results of this study, these patients were excluded. Therefore, the inclusion and exclusion criteria for this study were formulated as follows. Inclusion criteria: (1) T2DM, as defined according to a FBG concentration of ≥7.0 mmol/L (126 mg/dL), venous plasma glucose ≥11.1 mmol/L (200 mg/dL) 2 h after a glucose load challenge, or a random plasma glucose concentration ≥11.1 mmol/L (200 mg/dL), and age >18 years and (2) registered or permanent residence in the corresponding community for >1 year. Exclusion criteria: (1) acute metabolic disorder (such as diabetes ketoacidosis, hyperglycemia, or hypertonic state); (2) serious systemic diseases other than diabetes, such as severe cardiac/cerebrovascular disease or cancer; (3) eye diseases other than DR, such as severe cataract, glaucoma, or severe corneal opacity; (4) receiving clinically invasive treatment, including photocoagulation, ophthalmic surgery, or intraocular drug injection; and (5) no fundus examination was performed, or the quality of fundus photography was poor, which affected the diagnostic grade.

### 2.2. Measures

(1) A self-designed questionnaire was used to obtain data on the participants’ age, sex, disease course, and other basic information.

(2) Physical examination. Blood pressure was obtained using an electronic sphygmomanometer. Height and weight were measured with an ultrasonic instrument. Height, weight, waist, and hip circumference data of patients were measured accurately to 0.1 cm and 0.1 kg.

(3) Biochemical testing: After fasting for at least 8 h, fasting blood and urine samples were collected in the morning. Biochemical indicators included high-density lipoprotein, postprandial blood glucose (PBG), blood urea nitrogen, triglycerides, HbA1c, uric acid, total cholesterol, low-density lipoprotein, urinary microalbumin, creatinine, FBG, and glomerular filtration rate. FBG levels were determined using the glucose oxidase method, HbA1c levels using ion-exchange high-performance liquid chromatography, triglyceride and total cholesterol levels using enzyme colorimetry, and albumin and urinary creatinine using scattering turbidimetry. The urinary microalbumin to creatinine ratio was calculated and given as the albumin-creatinine ratio (ACR) (mg/g).

(4) The Canon CR-2 (Tokyo, Japan) fundus camera performed mydriasis-free fundus imaging for all subjects. The study number, sex, age, and disease course for each patient were entered into the fundus imaging control software. After placement in a dark room for 5 min, color photos of the 45° fundus’ posterior pole were taken in the non-mydriatic state, with the middle point between the optic disc and the macula as the center. Two photos were taken of each eye. Photos in JPG format (2592 × 1728 pixels) were sent to the ophthalmologist of the Sixth People’s Hospital Affiliated with Shanghai Jiao Tong University to diagnose and grade DR independently.

### 2.3. Statistical analysis

A total of 2,385 patients were identified, and the patients with abnormal indicators and missing data were excluded according to the inclusion and exclusion criteria.

SPSS 22.0 statistical software (IBM Corp., Armonk, NY, USA) was used for statistical analysis. T2DM patients were randomly divided into training and test sets in a ratio of 7:3. Continuous variables selected in section “2.2. Measures” are represented by mean ± standard deviation or median and quartile, and categorical variables are represented by frequency and proportion. Continuous variables were compared by *t*-test or Mann–Whitney *U*-test, and categorical variables were compared by chi-square test. For all analyses, *P* < 0.05 was considered statistically significant. The correlation between the variables in the training set was analyzed by a heatmap.

**FIGURE 1:**
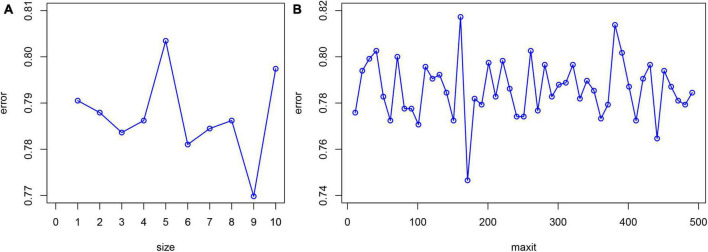
Hyperparameter adjustment of BPNN. The minimum prediction error occurred when the number of neurons equals to 9 **(A)** and the number of iterations is set to 170 **(B)**.

Statistical software package R (R Foundation for Statistical Computing, Vienna, Austria Version: 4.1.1) was used for feature filtering. The minimum absolute shrinkage selection operator (LASSO), extreme gradient boosting (XGBoost) model, a RF recursive feature elimination algorithm (RF-RFE), and back propagation neural network (BPNN) were used to filter the features of the training set. LASSO reduced the regression coefficient of the features with less influence to 0. These are then excluded from the model by setting the penalty coefficient. The variable with a non-zero regression coefficient has the strongest correlation with the response variable (39). A total of 10-fold cross-validation was performed using the glmnet package (version 4.1-2) to normalize and centralize the variables, and the best lambda values were selected to obtain a small number of important characteristic variables. The caret package (version 6.0-93), was used, where RF-RFE firstly trains the initial subset containing 23 features, then calculates the importance of 23 features, and finally obtains the classification accuracy of the current model through cross validation. The feature with the lowest priority is then removed, a new subset is obtained, and the above steps are repeated until the feature subset is empty. Finally, k-feature subsets and their corresponding classification accuracy are obtained, and we choose the best feature subset among them. XGBoost considers the processing of high-dimensional sparse data sets and missing values. It can automatically find the direction to split for samples with missing feature values. Feature sub-sampling was also introduced, which can reduce overfitting and the time required for training the model and predicting results (33). First, Matrix (version 4.0.0) was used to process the training set data and convert the data set into a sparse matrix. The mesh parameters of the model were set through the expand.grid function of the xgboost package (version 1.6.0.1) of R software, and brought into the train.xgb function to train the best parameters. Then, the best parameters obtained, such as nrounds = 75, max depth = 2, eta = 0.1, gamma = 0.5, colsample_ bytree = 1, min_ child_ Weight = 1, and subsample = 0.5, were brought into the xgb.train training model, set objective = “binary: logistic,” boost = “gbtree” for training, and then the importance diagram of variables was drawn through the xgb.plot.importance function. The basic idea for the BPNN is the gradient steepest descent method, and the core condition is to minimize the total network error by adjusting the weight value. The gradient search technique is used to minimize the error between the actual output value and the expected output value of the network (32). The nnet package (version 7.3-17) was used to adjust the hyperparameters through cross validation, and was iterated continuously to obtain the optimal model. Finally, the BPNN model in this study set a hidden layer, with 9 neurons and 170 iterations (Figure 1).

First, we analyzed the characteristics screened by the above four methods. We selected the characteristics existing in three or more methods as independent variables to construct the multivariable logistic regression Model I in which the presence or absence of DR is considered the corresponding dependent variable. Then, we created Model II with DR as the dependent variable. The risk factors determined by Mo et al. in the retrospective study were taken as the independent variables. These independent variables include age, systolic blood pressure (SBP), course of disease (Course), PBG, HbA1c, urinary creatinine, and urinary microalbumin (40).

Next, the performance of the two models was compared using nine evaluation indexes: accuracy, precision, recall rate, F1 score, calibration curve, balanced accuracy, area under receiver operating characteristic curve (AUROC), Hosmer-Lemeshow test, and the Net Reclassification Index (NRI). The AUROC is a commonly used indicator to evaluate binary classifiers (41). Calibration curves and the Hosmer-Lemeshow test were used to evaluate the accuracy of model fitting (40). The NRI was used to calculate the performance improvement scoring system of Model I relative to Model II (42). Finally, the best model was selected by evaluating and establishing the corresponding nomogram.

**FIGURE 2:**
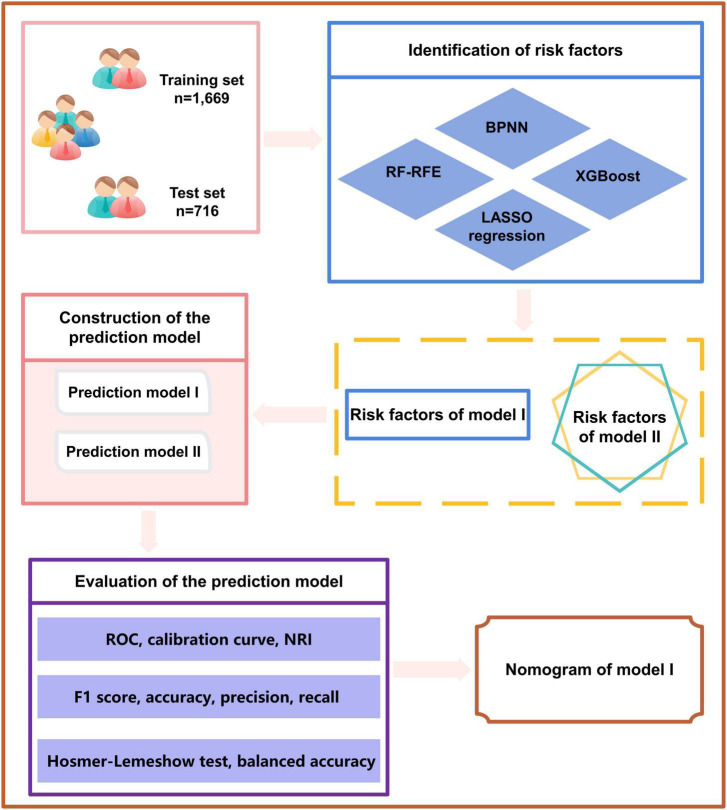
Study flowchart.

The tools used for all the data analysis included IBM SPSS statistics (IBM Corp. Released 2011. IBM SPSS Statistics for Windows, Version 20.0. Armonk, NY, USA: IBM Corp) and statistical software package R (R Foundation for Statistical Computing, Vienna, Austria Version: 4.1.1). A two-sided test was performed to conduct all statistical analyses, and the test level was α = 0.05. R language package was used for statistical analysis: Matrix (version: 4.0.0), glmnet (version:4.1-2), rms (version: 6.2-0), pROC (version: 1.17.0.1), rmda (version: 1.6), nricens (version: 1.6), magrittr (version: 2.0.1), fmsb (0.7.3), nnet (7.3-17), VRPM (version: 1.2), ggplot2 (version: 3.3.5), xgboost (version: 1.6.0.1), tidybayes (version: 3.0.2), readxl (version: 1.3.1), DynNom (version: 5.0.1), and plyr (version: 1.8.7). A flowchart of the study design is shown in Figure 2.

## 3. Results

### 3.1. Baseline characteristics

**FIGURE 3:**
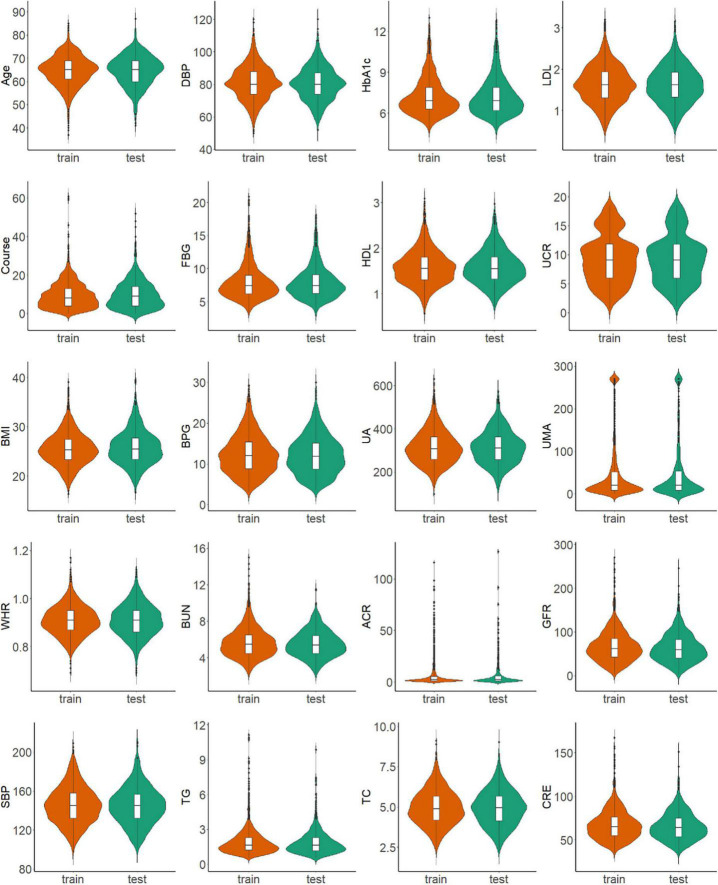
Distribution of continuous variables in the training and test sets.

**FIGURE 4:**
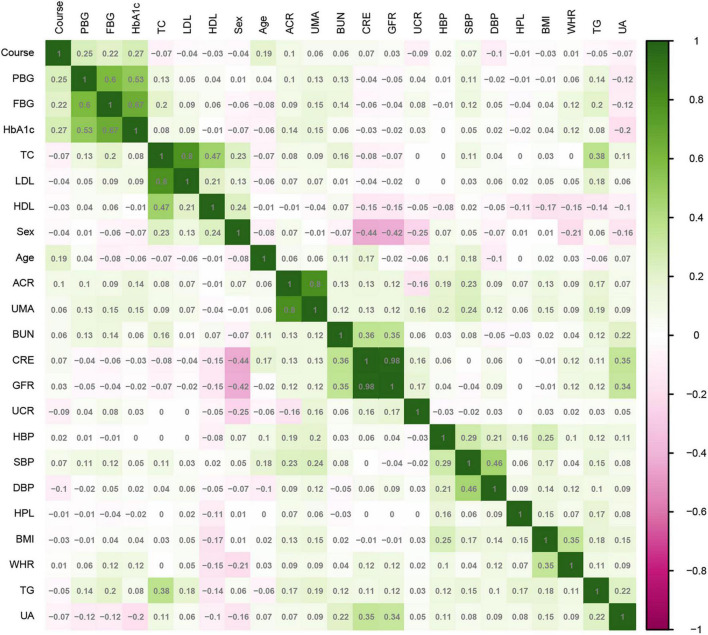
Correlation of variables in the training set.

The 2,385 T2DM patients were randomly divided into the training and test sets. In the training set, 1,669 participants were included, and 343 (20.55%) were diagnosed with DR. In the validation group, 716 participants were included, and 147 (20.53%) were diagnosed with DR. Table 1 shows the clinical and biochemical parameters for DR and non-DR in the training set. There was no significant difference in the basic characteristics between the training and test sets, except waist-to-hip ratio (*P* = 0.003) (Supplementary Table 1). The continuous variables for the two groups are represented by violin plots (Figure 3). Figure 4 shows the correlation between variables in the training set.

**TABLE 1:** Clinical features of the training set.

Variables	With DR (*n* = 343)	Without DR (*n* = 1326)	*P*-value
Age (year)	63.66 ± 6.56	64.70 ± 6.31	0.007
Sex (male/female)	160/183	574/752	0.264
Course (year)	11.00 (7.00, 16.00)	7.50 (4.00, 13.00)	<0.001
HBP (yes/no)	209/134	820/506	0.756
HPL (yes/no)	115/228	496/830	0.187
BMI (kg/m^2^)	25.68 ± 3.39	25.56 ± 3.29	0.530
WHR	0.92 ± 0.06	0.91 ± 0.06	0.008
SBP (mmHg)	148.01 ± 19.66	144.64 ± 18.99	0.004
DBP (mmHg)	80.23 ± 11.03	81.05 ± 10.09	0.190
FBG (mmol/L)	7.20 (6.10, 8.60)	8.20 (6.70, 10.70)	<0.001
PBG (mmol/L)	11.81 ± 4.52	14.11 ± 5.15	<0.001
BUN (mmol/L)	5.92 ± 1.72	5.54 ± 1.52	<0.001
TG (mmol/L)	1.81 ± 0.90	1.91 ± 1.15	0.135
HbA1c (%)	7.83 ± 1.66	7.06 ± 1.27	<0.001
HDL (mmol/L)	1.56 ± 0.37	1.59 ± 0.38	0.125
UA (μmol/L)	305.11 ± 74.82	316.17 ± 78.14	0.019
ACR (mg/g)	2.64 (1.07, 9.95)	2.04 (1.01, 4.96)	0.001
TC (mmol/L)	4.86 ± 1.14	4.95 ± 1.06	0.162
LDL (mmol/L)	1.59 ± 0.48	1.64 ± 0.45	0.072
UCR (umol/L)	8.99 ± 4.35	9.32 ± 4.12	0.185
UMA (mg/L)	24.00 (9.00, 71.00)	19.00 (8.00, 46.00)	0.007
CRE (μmol/L)	69.15 ± 25.10	67.22 ± 16.22	0.177
GFR (ml/min)	63.34 (42.80, 89.74)	62.11 (43.46, 85.43)	0.641

HBP, high blood pressure; HPL, hyperlipidemia; BMI, body mass index; WHR, waist-to-hip ratio; SBP, systolic blood pressure; DBP, diastolic blood pressure; PBG, postprandial blood glucose; FBG, fasting blood glucose; BUN, blood urea nitrogen; TG, triglyceride level; HbA1c, glycosylated hemoglobin A1c; HDL, high-density lipoprotein level; UA, uric acid; ACR, albumin-creatinine ratio; TC, total cholesterol; LDL, low-density lipoprotein level; UCR, urinary creatinine; UMA, urinary microalbumin; CRE, creatinine level; GFR, glomerular filtration rate.

### 3.2. Screening for risk factors

**FIGURE 5:**
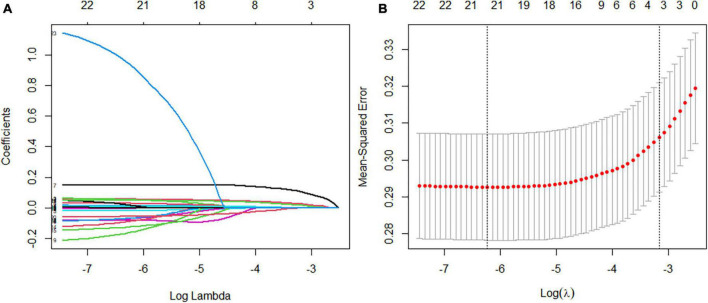
Feature selection using a least absolute shrinkage selection operator regression model. **(A)** The optimal parameter lambda filters out four variables with non-zero coefficients. **(B)** Partial likelihood deviation vs. log (lambda) is plotted after verification of the optimal lambda, and vertical dashed lines are drawn according to the 1-SE standard.

**FIGURE 6:**
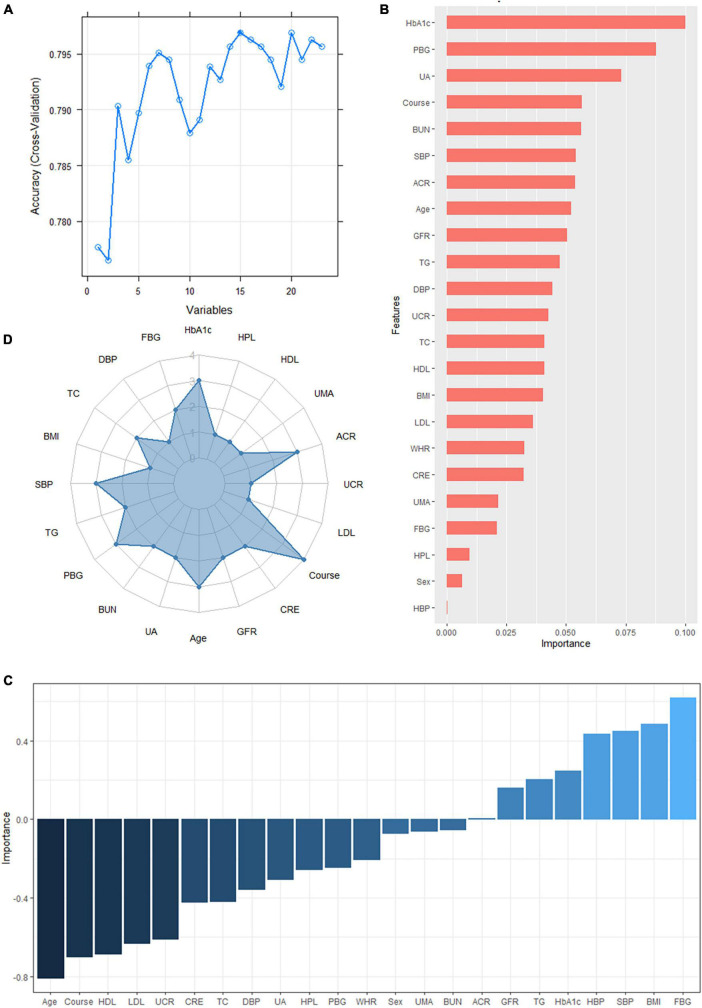
**(A)** The variables are screened using a random forest recursive feature elimination (RF-RFE). When the number of variables is 15, the model’s accuracy reaches the highest level. **(B)** Feature importance ranking of the extreme gradient boosting (XGBoost) algorithm. A longer bar means the variable has more influence on the outcome variable. **(C)** Feature importance ranking of the backpropagated neural network (BPNN). A higher bar indicates that the variable has a greater impact on the outcome variable; greater than zero means the variable is positively correlated with the outcome variable, and less than zero means the variable is negatively correlated with the outcome variable. **(D)** Display of the frequency of occurrence of variables screened by the least absolute shrinkage selection operator model, RF-RFE, and the top 10 features sorted by XGBoost and BPNN algorithm. Variables with three or more frequencies included HbA1c, course, postprandial blood glucose (PBG), age, systolic blood pressure (SBP), and albumin-creatinine ratio (ACR).

According to the literature search results and the results from the analysis of the questionnaire survey, 23 potential risk factors selected from the demographic characteristics, physical examination, and biochemical indexes were included in the LASSO regression method for analysis (Figure 5). Four characteristic variables with non-zero coefficients were obtained by LASSO regression analysis: HbA1c, Course, PBG, and ACR. The classification was most accurate when HbA1c, PBG, Course, FBG, glomerular filtration rate, creatinine level, ACR, uric acid, blood urea nitrogen, triglyceride level, TC, age, SBP, urinary microalbumin, and diastolic blood pressure (DBP) (15 feature variables) were incorporated into the RF classifier of the recursive feature elimination algorithm. The feature ranking of the XGBoost and BPNN algorithms is shown in Figure 6. The variables featured three or more times were screened, including HbA1c, Course, PBG, age, SBP, and ACR (Figure 6).

### 3.3. Construction of predictive models

The DR risk prediction Model I was established based on the six abovementioned predictors: HbA1c, Course, PBG, age, SBP, and ACR. A DR risk prediction Model II, including HbA1c, Course, PBG, age, SBP, urinary microalbumin, and urinary creatinine, was introduced.


$$M\,o\,d\,e\,l_{I}=-2.681+0.215\times H\,b\,A\,1\,c+0.057\times C\,o\,u\,r\,s\,e$$

+0.038×P⁢B⁢G-0.042×A⁢g⁢e+0.008×S⁢B⁢P

+0.015×A⁢C⁢R

$$M\,o\,d\,e\,l_{I\,I}=-2.465+0.221\times H\,b\,A\,1\,c$$

+0.056×C⁢o⁢u⁢r⁢s⁢e+0.037×P⁢B⁢G-0.043×A⁢g⁢e

+0.008×S⁢B⁢P+0.002×U⁢M⁢A-0.023×U⁢C⁢R

### 3.4. Evaluation of predictive models

**FIGURE 7:**
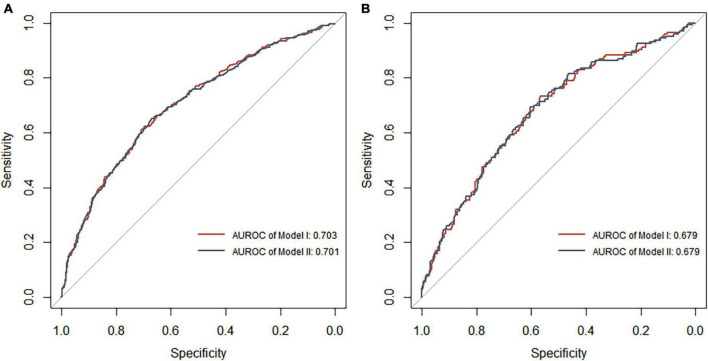
Receiver operating characteristic curve analysis of two DR risk prediction models in the training set **(A)** and test set **(B)**. The abscissa represents the false positive rate predicted by the model, and the ordinate represents the true positive rate predicted by the model.

**FIGURE 8:**
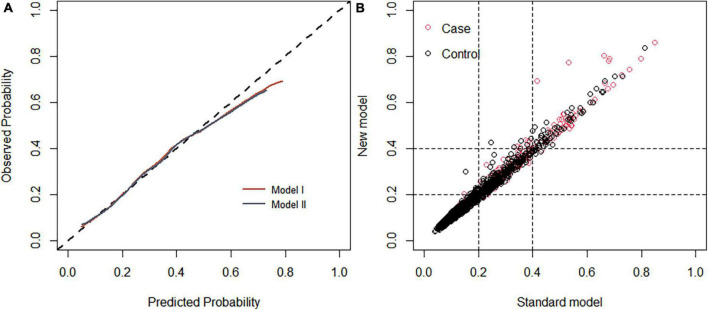
**(A)** Calibration curve of the diabetic retinopathy (DR) onset risk prediction model in the training set. The *x*-axis represents the predicted risk of developing DR, the *y*-axis represents the actual diagnosed DR, and the dashed diagonal line represents the perfect prediction from the ideal model. The solid line represents the model’s performance; the closer to the diagonal dotted line, the higher the model’s accuracy. **(B)** Net reclamation index (NRI)-based comparison. The NRI was 0.004 (95%: –0.068 to 0.132).

**FIGURE 9:**
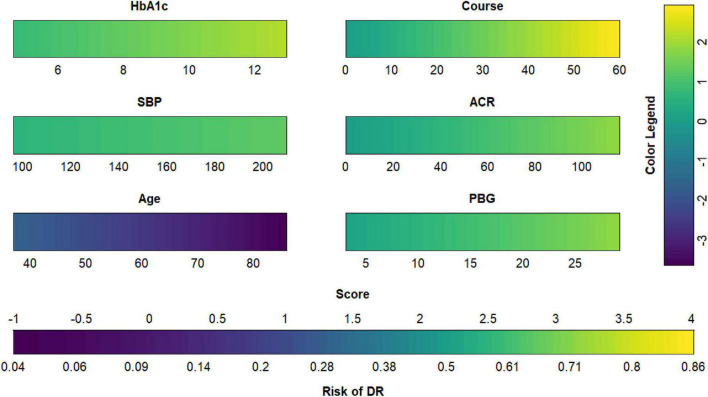
Diabetic retinopathy (DR) risk nomogram. Green indicates that the variable is a risk factor for DR, and blue indicates that the variable is a protective factor for DR. The darker the color, the greater the influence of the variable on DR.

**FIGURE 10:**
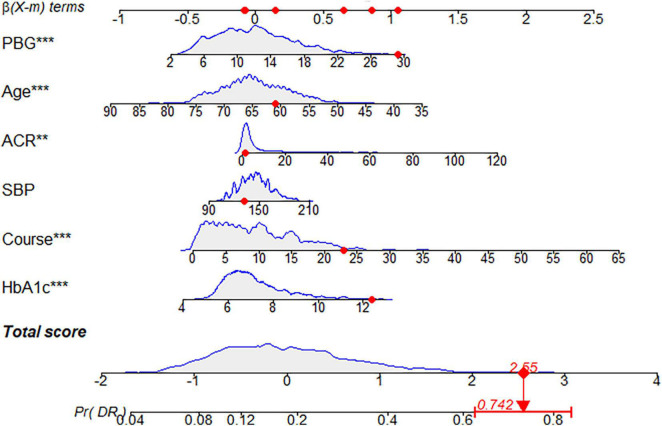
Dynamic nomogram. A dynamic nomogram based on Model I was created to predict the risk of developing diabetic retinopathy (DR) in type two diabetes mellitus (T2DM) patients. According to the patient’s albumin-creatinine ratio (ACR) (1.06 mg/g), systolic blood pressure (SBP) level (132 mmHg), age (61 years), postprandial blood glucose (PBG) level (29.3 mmol/L), course (23 years), and glycosylated hemoglobin type A1c (HbA1c) (12.4%), the predicted probability of DR was 0.742. Therefore, the patient had an 74.2% chance of developing DR.

Each model was evaluated by accuracy, precision, recall, F1 score, and balanced accuracy, as shown in Table 2. The AUCs of Model I and Model II in the training set were 0.703 and 0.701, respectively; the AUCs of Model I and Model II in the test set were 0.679 and 0.679, respectively (Figure 7). The calibration curve was used to correct prediction Models I and II, respectively (Figure 8). The results of the Hosmer-Lemeshow test showed that the *P*-values for Models I and II in the training set were 0.887 and 0.760, respectively. The NRI index was 0.004, and thus Model I was more discriminative than Model II (Figure 8). After evaluation, Model I was determined to be the optimal predictive model. A dynamic nomogram was built to calculate (Figure 9) and visualize the risk of developing DR in Chinese T2DM patients (Figure 10). Concurrently, the corresponding dynamic nomogram application program was developed.^1^

**TABLE 2:** Different indicators for evaluating the effectiveness of DR risk prediction models.

Model	Accuracy	Precision	Recall	F1-score	Balanced accuracy
Model I	0.796	0.571	0.035	0.066	0.514
Model II	0.796	0.550	0.032	0.060	0.513

## 4. Discussion

In this cross-sectional study based on T2DM patients in the community, we developed a practical and sufficiently discriminative risk prediction model. The corresponding nomogram can help medical workers identify the risk of DR in T2DM patients for its prevention and treatment. Furthermore, based on this result, practitioners can prioritize which patients should undergo fundus imaging diagnosis. Compared with the Model II proposed by Mo et al., which has been used to predict disease risk using 7 indicators, our model obtained a good performance in predicting DR, but Model I only used 6 indicators. Model I has a 0.1% higher area under the curve and a higher discriminative power than Model II (NRI = 0.004). Model I and Model II share some DR risk factors, including HbA1c, course, PBG, age, and SBP, which have also been confirmed in other relevant studies (25, 40, 43).

The occurrence of DR is closely related to the course of hyperglycemia and diabetes (7, 25, 44). Both HbA1c and PBG are general indicators of blood sugar. HbA1c mainly reflects the average blood sugar level in the last 2–3 months, and the latter indicates the degree of blood sugar fluctuations (43). HbA1c is a key parameter for diabetes control, and excessive blood glucose levels can produce oxidative stress and micro-inflammation, which are important mechanisms leading to T2DM and related complications (45–47). The presence and severity of DR are positively correlated with HbA1c (48). The hyperglycemic environment of diabetes leads to activation of the polyol metabolic pathway, an increased level of oxidative stress in retinal tissue, and damage to the retinal capillary endothelial cells, causing retinal ischemia and hypoxia. This is followed by the destruction of the blood-retinal barrier and the formation of new blood vessels, resulting in the pathological changes seen in DR (49). Longer disease duration may imply a longer duration of retinal toxicity induced by high glucose levels, increased activation of protein kinase C, and increased vascular endothelial growth factor (VEGF) activity, thereby promoting the development of DR (50). Ding and Wang analyzed 35 epidemiological studies and reported that the DR prevalence rate increased significantly with the increase in the disease course. The DR incidence rate of patients with DM < 10 years is 21.1%, and that of patients with T2DM >20 years is 76.3% (51). Currently, most people with T2DM can prevent and control the worsening of the disease through lifestyle changes, including eating a balanced diet, maintaining physical activity, and not smoking or drinking alcohol (52, 53). In addition, metformin, which can reduce FBG levels and reduce morbidity and mortality, is an effective means of T2DM prevention and treatment (54).

We found that hypertension is closely associated with DR development, which has been confirmed in previous studies (40, 55, 56). A large population-based cross-sectional survey in China found that elevated blood pressure promotes DR development, and lower blood pressure slows its progression (48). In another study, patients with T2DM who had hypertension for >10 years were more than twice as likely to develop DR than those without hypertension (57). Hemodynamic changes, such as impaired autoregulation, increased blood perfusion caused by hypertension independent of hyperglycemia, and stimulated upregulation of VEGF expression in retinal endothelial cells and ocular fluid, all affect the development of DR (58, 59). An epidemiological study of Latino populations by Varma et al. reported that each 20-mmHg increase in SBP was related to a 1.26-fold increase in the risk of developing DR. Karoli et al. revealed that hypertension and elevated SBP were present in DR patients. Individuals with elevated SBP were at increased risk of developing retinopathy (60). The United Kingdom Prospective Diabetes Study emphasized that strict blood pressure control can effectively delay the development of DR. After 4.5 years of follow-up, patients with a blood pressure control target of 150/85 mmHg developed fewer retinal microvessels than those with a control target of 180/105 mmHg. The pathological changes characteristic of DR, such as tumor, hard exudate, and cotton wool spots, are less likely. The number of retinal laser photocoagulation treatments in patients with strict blood pressure control is also lower (61). Hypertension promotes arteriosclerosis, resulting in insufficient blood supply to retinal tissue, aggravating retinal ischemia and hypoxia, and accelerating DR progression. Strict control of blood pressure can reduce the progression of DR (7). International experience shows that community prevention and treatment is the most effective way to control hypertension and its rising incidence (7, 62). Community health service institutions need to pay attention to patients with hypertension and include groups at high risk of hypertension in health management plans. Specific measures include: (1) provide health education to groups at high risk of hypertension to promote understanding of the harm of hypertension, advocate a reasonable diet, promote appropriate exercise, maintain a positive outlook, and avoid prolonged mental stress (63); and (2) regular follow-up and examination of groups at high risk of hypertension to achieve early detection, diagnosis, and treatment.

This study found that the risk of DR was inversely related to the age of T2DM onset; that is, the older one is at the T2DM onset, the lower the risk of DR, which is consistent with the results of previous studies (40, 64). The mechanism of the high risk of DR in patients with early onset T2DM is unclear, and it may be a combination of factors. Some studies have found that VEGF activity may vary according to age, with higher expression in younger patients with causative factors. In addition, younger patients are less concerned about their health than older patients, and their treatment compliance and glycemic control are often not as good, which may aggravate the development of their diabetic complications (65). Therefore, equal attention should be paid to managing young and elderly patients in community DR prevention and treatment to strengthen the health education and management of young patients, promote an interest in their personal health, and encourage them to actively cooperate with treatment.

We found that the risk of DR increased as ACR increased. A population-based, prospective 10-year follow-up study found that the ACR was a risk factor for sight-threatening DR and diabetic macular edema, with hazard ratios of 2.448 and 2.432, respectively (66). A survey of 28,344 T2DM patients by Rodríguez-Poncelas et al. revealed that the prevalence of DR increases with ACR levels and that a significant effect on DR begins when the ACR is >10 mg/g (67). Studies have also shown that DR is related to the severity of diabetic nephropathy and that the two influence each other (68–70). A cross-sectional survey of 971 Korean T2DM patients found that DR was significantly associated with renal impairment and overt nephropathy. It was recommended that when physicians diagnose T2DM patients with DR, they should promptly evaluate the patient’s renal function. This may be due to the similar pathogenesis of DR and diabetic nephropathy, including hyperglycemia-induced oxidative stress, accumulation of glycation end products, increased reactive oxygen species, abnormal activation of protein kinase C, and abnormal renin-angiotensin system activation (68, 71, 72). ACR is a common index to assess renal function and was shown to be a predictor of DR in this study (68).

It is an important public health problem to reduce or delay the occurrence of DR based on standardized management of T2DM patients (73). Due to the limitation of social and medical resources, lower examination rates delay the DR diagnosis and treatment (74, 75). Studies have shown that the cost of community DR screening and subsequent treatment is much lower than that of treatment in the absence of screening (4, 76, 77). Therefore, it is necessary to improve further the family doctor system and the community chronic disease management model and promote the integration of community chronic disease prevention and treatment. We have developed an effective nomogram to identify more specific and sensitive biomarkers, which can help general practitioners detect patients at high risk of DR early, develop personalized health management plans, and reduce the risk of poor vision and blindness in patients with diabetes. Moreover, the nomogram may assist in reducing the morbidity rate and further improve chronic disease management in Chinese communities. First, general practitioners should screen high-risk patients using the DR risk prediction model. Intervention measures for high-risk patients to improve their awareness of DR and self-management capabilities should be implemented to control the disease and improve their quality of life. These interventions should include early medical treatment, regular monitoring, medication as prescribed, and diet and exercise control to effectively reduce blood sugar and blood pressure levels (73). Second, when managing T2DM, community health service organizations should regularly monitor T2DM indicators and closely related diseases, such as hypertension and diabetic nephropathy. This will allow for the timely detection of the possible occurrence of DR in T2DM patients and allow for interventions to improve the effectiveness of DR control. Finally, the burden on general hospitals can be relieved by conducting the initial screening and monitoring in community hospitals. Utilizing specialized hospitals for symptomatic treatment can improve the efficiency and quality of patients’ medical care and reduce the economic burden on society, families, and individuals.

This study is based on the data collected by the Chinese community population, with sufficient sample size and high local applicability. Few predictors, which were easily collected, were selected with certain accuracy through a variety of machine learning methods. In particular, a DR risk prediction model proposed by Mo et al. (40), namely Model II, was introduced for multiple evaluations in order to evaluate the effectiveness of Model I by using the same sample. The research results showed that the prediction ability of the Model I built in this study was better than that of the Model II established by Mo et al. (40), and the prediction factors were lesser in our study.

In this study, only physical factors were taken in consideration, but other factors related to lifestyle, including physical activity and diet, may have an important influence on DR. In the future, social-life-related factors having an influence on the disease should be included to improve the prediction ability of the model. In addition, a dynamic monitoring system should be established to track the dynamic changes of residents’ indicators in a timely manner to understand the development and progress of diseases, to verify the effectiveness of the model, and to amend the model.

Our study had some limitations. First, this was a cross-sectional study, and we only observed the static state of the indicators in 2,385 community T2DM patients. If the patients were followed-up to obtain the dynamic changes in various indicators, the accuracy of the nomogram should have improved. Second, the population of this study was concentrated in Shanghai. Although the stability of our prediction model had been confirmed in this population, it has not been verified in other regions or countries. Therefore, our prediction model needs to be evaluated in a broader range of populations.

## 5. Conclusion

This study retrospectively analyzed T2DM patients using LASSO regression analysis, an XGBoost model, an RF-RFE, and a BPNN to screen DR risk factors and construct a DR incidence risk prediction model based on logistic regression analysis. Compared with Model II, Model I, including HbA1c, course, PBG, age, SBP, and DBP (six characteristic variables), has advantages in accuracy and discrimination. By calculating a patient’s DR risk through the nomogram of Model I, general practitioners can have a reference for the screening and early diagnosis of DR in patients with T2DM and reduce the risk of DR. This will improve the quality of life of these patients as well as the management of chronic diseases in the community.

## Data availability statement

The raw data supporting the conclusions of this article will be made available by the authors, without undue reservation.

## Ethics statement

This study has been approved by the Medical Ethics Committee of Longhua Hospital, Shanghai University of Traditional Chinese Medicine (approval number: 2017LCSY069). The patients/participants provided their written informed consent to participate in this study.

## Author contributions

AZ designed the study. HA, JZ, and AZ obtained the data. HP, JS, and XL sorted out the data. JS and HP analyzed, interpreted, and discussed the data. HP wrote this manuscript. All authors approved the final version of this manuscript.

## Footnotes

1
https://doctorpan.shinyapps.io/DyNomapp/
