# Supplementary material for: A risk prediction model for type 2 diabetes mellitus complicated with retinopathy based on machine learning and its application in health management
Source: Front Med (Lausanne). 2023 Apr 27;10:1136653. doi: 10.3389/fmed.2023.1136653 (PMC10172657; doi:10.3389/fmed.2023.1136653)
Supplement: Supplementary file 1 [file Table_1.DOCX]

| Variables | Training set (n = 1669) | Test set (n = 716) | P value |
| --- | --- | --- | --- |
| Age (year) | 64.53 ± 6.42 | 64.61 ± 6.54 | 0.776 |
| Sex (male/female) | 718/951 | 302/414 | 0.704 |
| Course(year) | 9.00 (4.00, 14.00) | 8.00 (4.00, 14.00) | 0.668 |
| HBP (yes/no) | 1051/618 | 429/287 | 0.159 |
| HPL (yes/no) | 623/1046 | 259/457 | 0.592 |
| BMI (kg/m2) | 25.54 ± 3.34 | 25.59 ± 3.32 | 0.718 |
| WHR | 0.91 ± 0.06 | 0.90 ± 0.06 | 0.003 |
| SBP (mmHg) | 145.35 ± 19.15 | 145.31± 19.35 | 0.962 |
| DBP (mmHg) | 81.03 ± 10.19 | 80.48 ± 10.42 | 0.234 |
| FBG (mmol/L) | 7.96 ± 2.57 | 7.84 ± 2.47 | 0.272 |
| PBG (mmol/L) | 12.33 ± 4.79 | 12.17 ± 4.64 | 0.457 |
| BUN (mmol/L) | 5.64 ± 1.57 | 5.59 ± 1.61 | 0.497 |
| TG (mmol/L) | 1.93 ± 1.14 | 1.84 ± 1.07 | 0.097 |
| HbA1c (%) | 7.25 ± 1.40 | 7.20 ± 1.32 | 0.445 |
| HDL (mmol/L) | 1.58 ± 0.37 | 1.57 ± 0.38 | 0.463 |
| UA (μmol/L) | 313.53 ± 78.54 | 314.69 ± 77.62 | 0.741 |
| ACR (mg/g) | 2.14 (1.05, 5.34) | 2.09 (1.05, 5.91) | 0.702 |
| TC (mmol/L) | 4.96 ± 1.08 | 4.90 ± 1.07 | 0.170 |
| LDL (mmol/L) | 1.64 ± 0.45 | 1.62 ± 0.46 | 0.581 |
| UCR (umol/L) | 9.34 ± 4.16 | 9.08 ± 4.19 | 0.161 |
| UMA (mg/L) | 24.00 (9.00, 71.00) | 19.00 (8.00, 46.00) | 0.845 |
| CRE (μmol/L) | 67.00 ± 17.42 | 67.37 ± 20.23 | 0.674 |
| GFR (ml/min) | 66.31 ± 35.88 | 66.99 ± 42.17 | 0.708 |

Supplementary table 1 basic characteristics between the training and test sets
